# Reconstructive Options for Oncologic Posterior Trunk Defects: A Review

**DOI:** 10.3389/fonc.2016.00051

**Published:** 2016-03-08

**Authors:** Björn Behr, Johannes M. Wagner, Christoph Wallner, Kamran Harati, Marcus Lehnhardt, Adrien Daigeler

**Affiliations:** ^1^Department of Plastic Surgery, BG University Hospital Bergmannsheil Bochum, Bochum, Germany

**Keywords:** plastic surgery, posterior trunk, back, reconstruction, flap

## Abstract

After oncological tumor resections at the back, large defects can remain that depending on the size and location may represent reconstructive challenges to plastic surgeons. Flap selection includes the entire armamentarium of coverage, including transposition flaps, perforator flaps, pedicled muscle flaps, and free flaps. Most defects can be closed and reconstructed with local or pedicled muscle flaps. In our hands, sufficient closure could be obtained with all techniques, except the latissimus dorsi turn-over flap. Thereupon, an algorithm for closure of posterior trunk defects related to the anatomical region is proposed.

## Introduction

Soft-tissue defects of the posterior trunk may represent challenges for reconstructive surgeons. Reasons include shortages of both, reliable axial pattern flaps for local tissue transfer and recipient vessels for microsurgical reconstruction (1). Upon the various causes such as trauma, congenital malformations, spine surgery, or radiation ulcers, malignant skin or soft-tissue tumors may also put the back at risk for soft-tissue defects. At first, general surgical principles such as proper debridement and adequate preparation of the wound have to be performed. Depending on the characteristics of the tumor, single-stage or sequential operations may be indicated. Eventually, vacuum-assisted wound conditioning may be performed. In case, spinous processes are exposed, they may be readily removed in order to avoid any perforations or subsequent pressure sores after soft-tissue coverage. Besides therapeutic concepts such as secondary healing or skin grafts may only be of partial benefit to these patients (2). Given the relative lack of elasticity as well as a shortage of potential microsurgical recipient vessels, the back offers special challenges to the reconstructive surgeon (3). Nevertheless, the dorsal trunk hosts several muscles that may be transferred as pedicled flaps such as M. latissimus dorsi or M. trapezius flaps. A popular fasciocutaneous option is the parascapular flap; though given their cephalic pedicle, they are not useful for reconstruction of the lower back. Moreover in selected cases, free flaps with vein grafts or loops may be utilized. More recent trends involve the application of fasciocutaneous perforator flaps since the posterior trunk involves abundant perforators. Of note, the concept of perforator-based soft-tissue reconstruction was first described for defect coverage at the back (4). This article describes and illustrates the different soft-tissue reconstruction techniques for the posterior trunk.

## Transposition Pattern and Perforator Flaps

**Figure 1:**
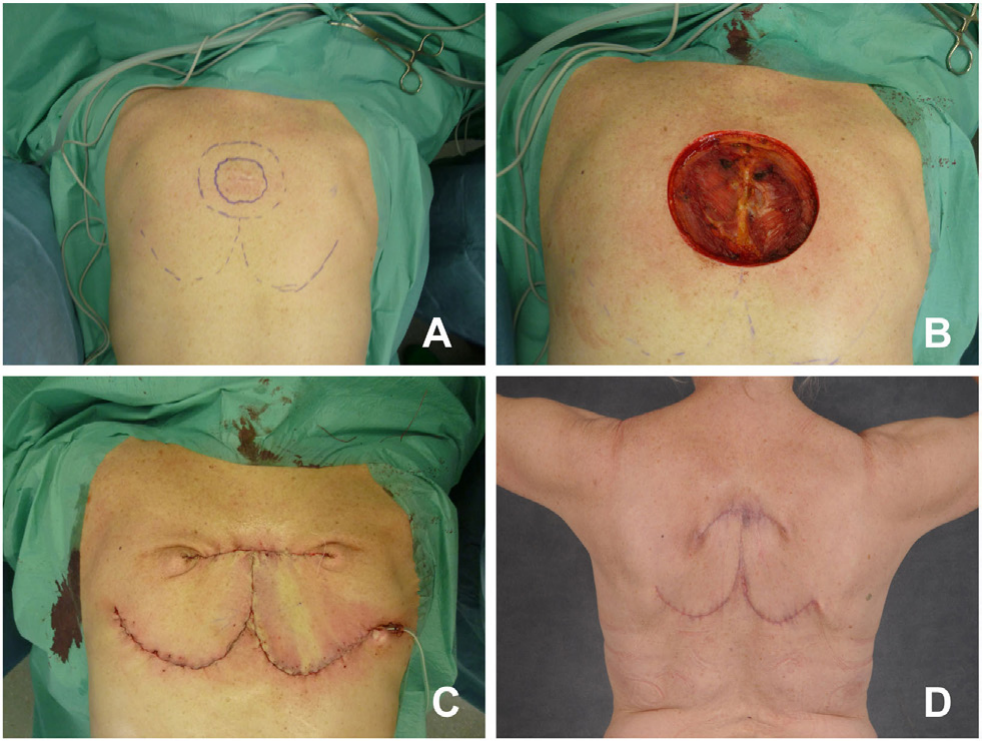
**Bilateral rotation flap**. **(A)** Patient with dermatofibrosarcoma protuberans at the upper thorax. Preoperative markings, two bilateral rotational flaps are planned from caudal. **(B)** Intraoperative status after resection. **(C)** Status after rotation of the flaps and wound closure. **(D)** Postoperative result.

Given the large area and abundance of perforating vessels in the posterior trunk, this anatomic region offers multiple options for plastic-surgical reconstruction by means of random pattern and especially perforator flaps (5). If established principles such as a proper width–length ratio in random-pattern flaps, as well as the right angiosome size in perforator flaps are considered, these flaps are safe and can cover many defects that occur (Figure 1). It has to be stressed though that utilization of these flaps is highly dependent on the quality of the surrounding tissue, which might be compromised due to radiation or scarring. However, with these techniques, the underlying muscles are conserved and may be utilized in later reconstructions, if necessary. For the operative planning, a handheld Doppler or power Doppler to track the route of the perforator vessel is advisable. Moreover, combination of both techniques is possible. For instance, a perforator can be integrated into a transposition flap in order to design it larger and safer.

Options for pedicled fasciocutaneous flaps at the back include parascapular and scapular flaps. These flaps are supplied by the circumflex scapular artery and can cover defects of upper- and mid-thoracic area of the back. Similar as in a free flap procedure, the pedicle can be dissected up to the level of the subscapular artery and vein; however, this is rarely necessary. Dimensions of parascapular flaps can reach up to 15 cm × 40 cm; therefore, they can be considered larger than scapular flaps.

**Figure 2:**
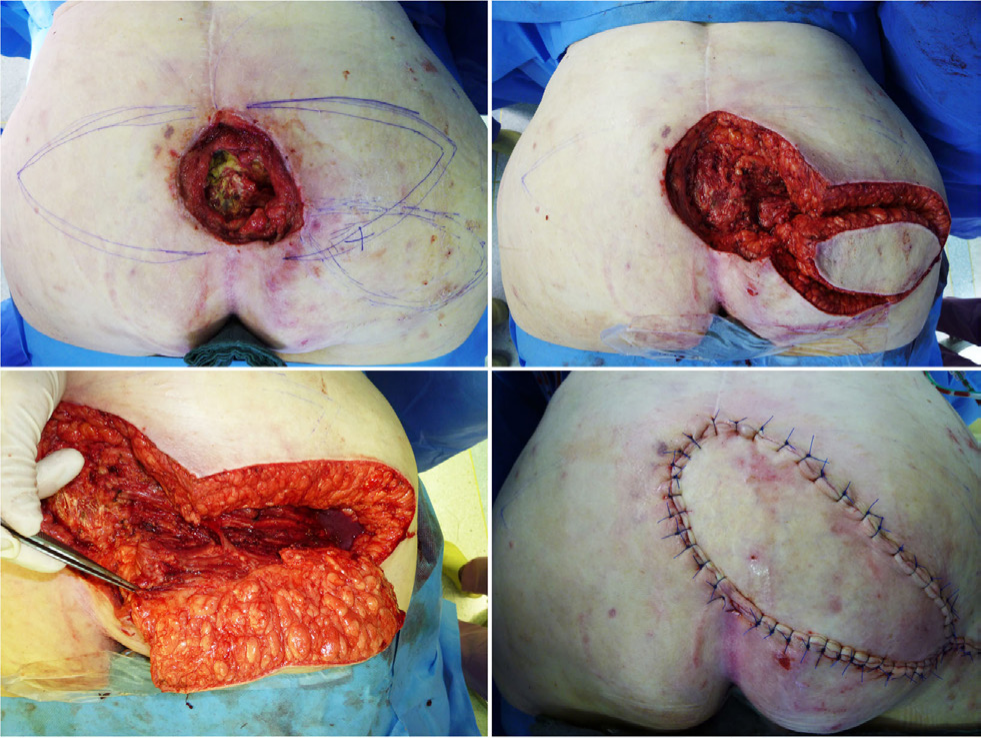
**S-GAP perforator flap for coverage of the sacrum**. **(A)** Status after debridement and planning of the flap. **(B)** Flap before rotation. **(C)** Identification and preparation of the perforator. **(D)** Postoperative result.

Moreover, gluteal perforator flaps can be useful in coverage of sacral defects (6). For instance, the *superior gluteal artery flap* (S-GAP) can be dissected without major damages to the gluteus maximus muscle (Figure 2). Finally, lumbar- or intercostal artery-based perforator flaps may be utilized in this anatomical region (7).

## Supraclavicular Flaps

Supraclavicular flaps that can also be raised as island flaps represent an interesting fasciocutaneous option for soft-tissue coverage of the dorsal neck region. As indicated by its name, it is raised from the supraclavicular region based on the transverse cervical artery and has dimensions up to 22 cm in length and 10 cm in width (8). Even in complicated surgical conditions, these flaps can provide reliable defect coverage in the anterior and posterior neck region (9).

## Trapezius Flap

**Figure 3:**
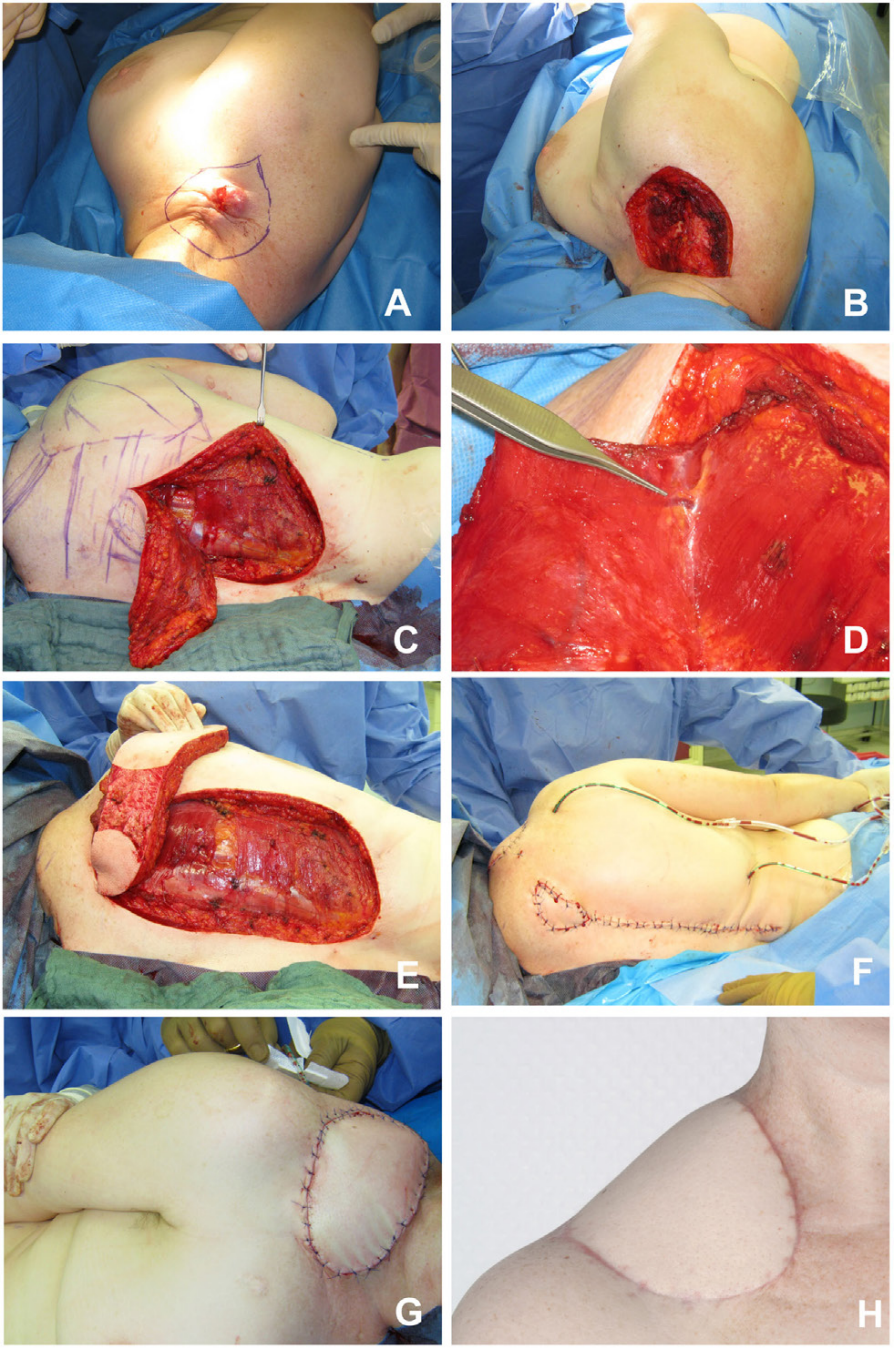
**Trapezius flap for coverage of the right shoulder**. **(A)** Cutaneous metastasis of breast cancer at the right shoulder. **(B)** Status after resection. **(C)** Preparation of the myocutaneous trapezius muscle from caudal to cephalic. **(D)** Identification of the deep branch of the cervical artery and vein. **(E)** Transposition of the myocutaneous flap. **(F,G)** Intraoperative insert of the flap. **(H)** Postoperative result.

The trapezius muscle, the superficial muscle of the neck and upper-thoracic region is an excellent option to cover defects in the upper thorax and neck region (10). It is traditionally considered a class 2 muscle according to (11) (one dominant and several minor vascular pedicles), although others have noted two dominant vessels for separate portions of the muscle. The inferior part of the trapezius muscle is supplied by the dorsal scapular artery (a deep branch of the cervical artery), whereas the transverse part is supplied by the superficial cervical artery. It extends from the external occipital protuberans to the 12th thoracic spine. It is up to 34 cm × 18 cm in size and can be dissected as a muscle-only or myocutaneous flap. The muscle can be dissected from caudal to cephalic and can be rotated into defects of the upper posterior trunk and dorsal neck. Moreover, they can be utilized as advancement flaps or turn-over flaps (Figure 3) (12, 13). In that respect, it has to be emphasized that good results can be obtained when the skin island does not exceed the muscle for more than 1 cm (14).

## Latissimus Dorsi Flap

**Figure 4:**
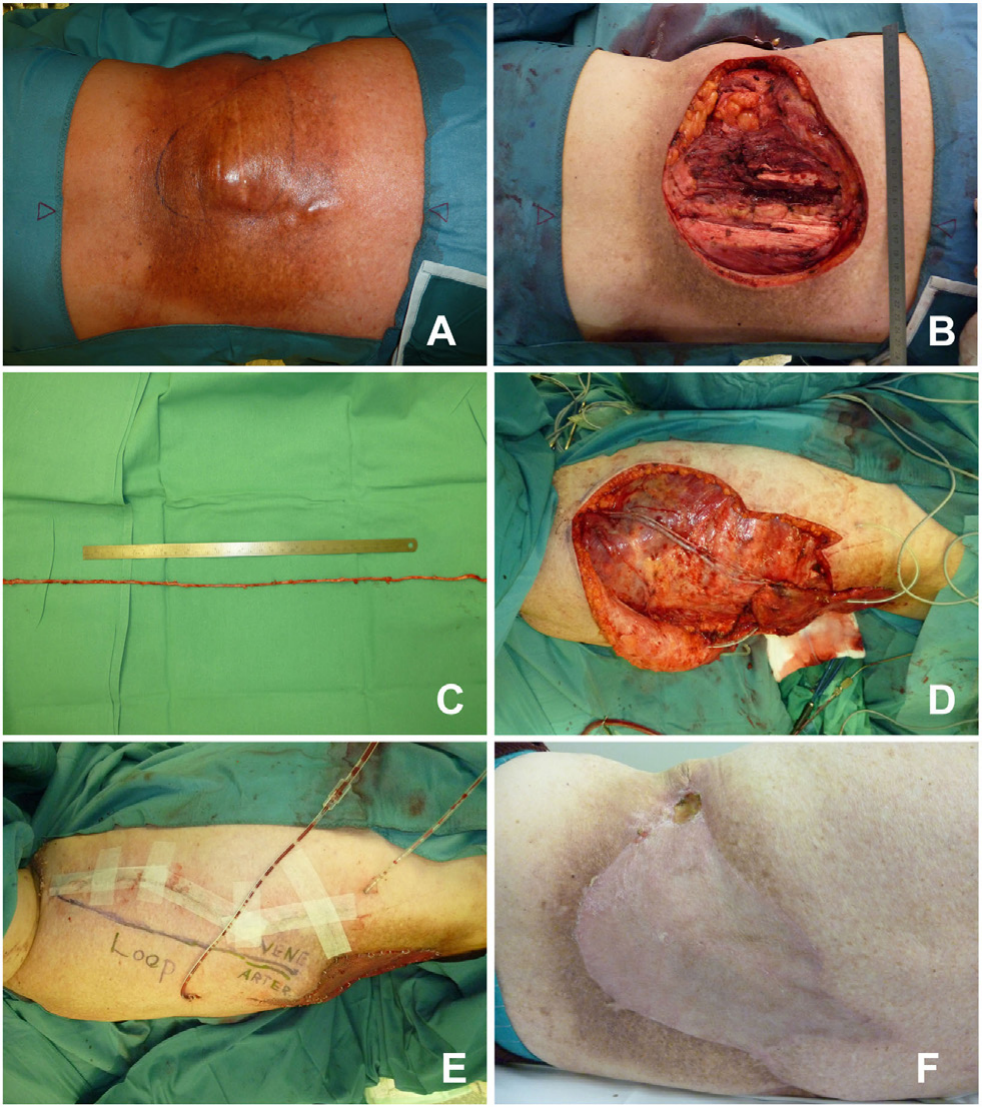
**Latissimus dorsi flap with vein transposition**. **(A)** NOS sarcoma in the left lumbar region. **(B)** Resected tumor. **(C)** Great saphenous vein graft with 30 cm ruler for comparison purposes. **(D)** Latissimus dorsi muscle transferred with two interposed veins. **(E)** Operative situs after skin closure. **(F)** Postoperative result.

The latissimus dorsi muscle flaps offers great variety and options to cover large defects in the mid-thoracic and upper-thoracic posterior trunk. It can be raised up to 30 cm × 40 cm in size and may be transferred as a muscular (eventually with additional skin grafts) or myocutaneous flap. The latter option makes postoperative monitoring considerably easier. It origins at the thoracical spinous processes, inferior ribs, and iliac crest. The latissimus dorsi muscle inserts at the intertubercular groove of the humerus. Its dominant vascular pedicle is the thoracodorsal artery, which is part of the scapular vascular system, whereas the non-dominant pedicles origin from intercostal and lumbar arteries. It is therefore a class 5 muscle according to the popular classification of Mathes and Nahai; thus, survival of the flap may also be based on the non-dominant pedicles (15), which would allow utilization of this flap as a “reverse” flap in order to cover contralateral or more caudal defects. However, in certain instances such as previous spinal surgery, these lumbar perforators may not exist anymore; hence, preoperative Doppler control is highly suggested in these instances. In our experience, the reverse latissimus dorsi muscle produced inconsistent results; therefore, it is no longer part of our armamentarium. An alternative for more caudal defects is the transposition of vein grafts to increase the reach of latissimus dorsi muscle flaps (Figure 4) (16).

## Paraspinous Muscle Flap

An additional option for small defects in the paravertebral region is the paraspinous muscle flap (17). Paraspinous muscles are located in the lumbar region up to the 10th thoracic vertebrae and are typically utilized as bipedicled turn-over flaps. Muscles are dissected off the transverse processes of the vertebrae and advanced medially; thus, wound in the perivertebral region can be closed with two pedicled paraspinous flaps.

## Pull-Through VRAM-Flaps

In certain conditions, such as sacral wound coverage after abdominoperineal resection, pull-through vertical rectus abdominis muscle (VRAM) flaps may be utilized (18). Here, the rectus abdominis muscle is pedicled on the inferior epigastric artery and the flap (muscle including skin island) is transpelvically pulled through to the sacrum.

## Gluteus Maximus Flap

**Figure 5:**
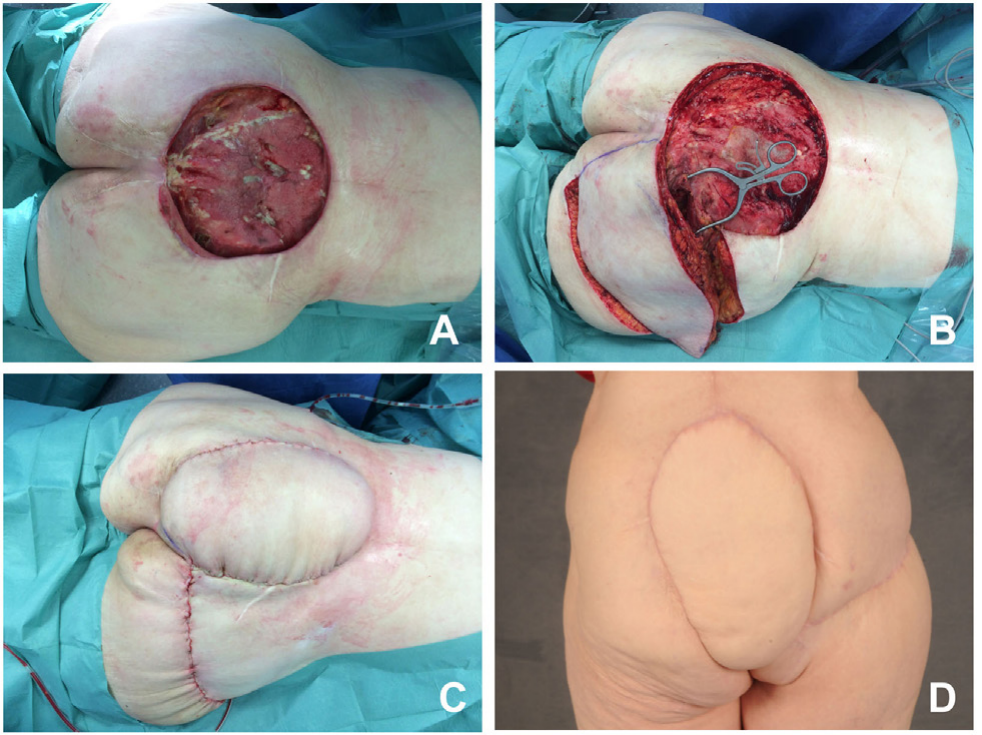
**Gluteus maximus flap**. **(A)** Large defect in the sacral region. **(B)** Myocutaneous gluteus maximus flap before transposition. **(C)** Operative situs after skin closure. **(D)** Postoperative result.

Besides utilization of gluteal perforator flaps, defects in the sacral region can likewise be reconstructed with the gluteus maximus muscle flap (Figure 5). This is a type 3 muscle with two dominant pedicles (superior and inferior gluteal arteries). Especially, the superior gluteal artery is a useful pedicle for the advancement or turn-over gluteus maximus muscle flaps for sacral defects (19).

## Microsurgery and Options for Recipient Vessels

**Figure 6:**
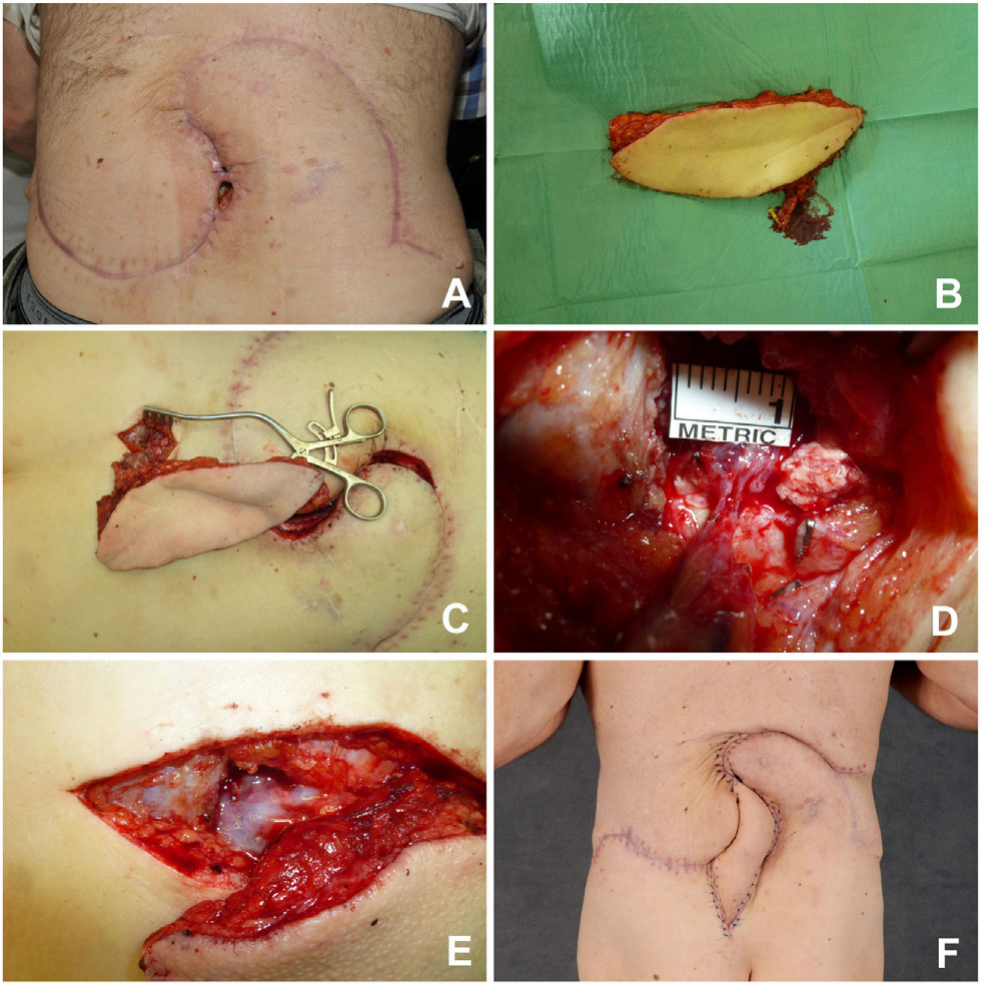
**Fasciocutaneous infragluteal free flap (FCI)**. **(A)** Remaining lumbar defect after two rotational flaps. **(B)** FCI flap with pedicle. **(C)** Anastomosis of the flap to a lumbosacral perforator. **(D)** Close-up view, revealing the small caliber of the recipient vessel. **(E)** Fibrin glue protection of the pedicle. **(F)** Early postoperative result.

In rare instances where defects of the posterior trunk can not be managed by local or pedicled flaps, microvascular free flaps have to be utilized. Although there are virtually no limits in terms of potential donor sites in the selection of free flaps, the actual choice for recipient vessels might prove to be challenging. One potential recipient vessel is the superior gluteal artery at the buttock (20). Moreover, microvascular free flaps may also be anastomosed to the fourth lumbar artery, lateral of the sacrospinal muscle (Figure 6). In case these options are not feasible, transpositions of vein grafts are necessary. Few et al. have reported about their experience in the “hostile” back, defined as a defect larger than 200 cm^2^ in size, previous radiation therapy, fulminant infections, or exposed hardware (21). In four of their patients, defects were closed with free latissimus dorsi flaps or VRAM flap with interposition of vein grafts. For this purpose, the great saphenous vein may be readily utilized. Of note, there is no need to reposition the patient intraoperatively when operating latissimus dorsi flaps with vein grafts.

## Special Considerations

When operating at the posterior trunk, there are a couple of obstacles to consider, which should be taken into consideration preoperatively. For instance, leakage of cerebrospinal fluid should prompt an interdisciplinary approach with neurosurgery to adequately reconstruct the dura. Another problem that may be encountered is exposed spinal hardware. In case fusion is noted on CT-scans, the hardware may be removed; however, in all other patients, instruments may remain *in situ* (22, 23). Finally as previously noted, radiation of the defect area in the medical history may further bedevil the situation and preclude local tissue transfer. Importantly, ulcers in these radiated areas may contain neoplastic processes, such as squamous cell carcinoma, which should be histologically excluded.

## Approach for Selection of the Flap Related to the Anatomic Region

**Figure 7:**
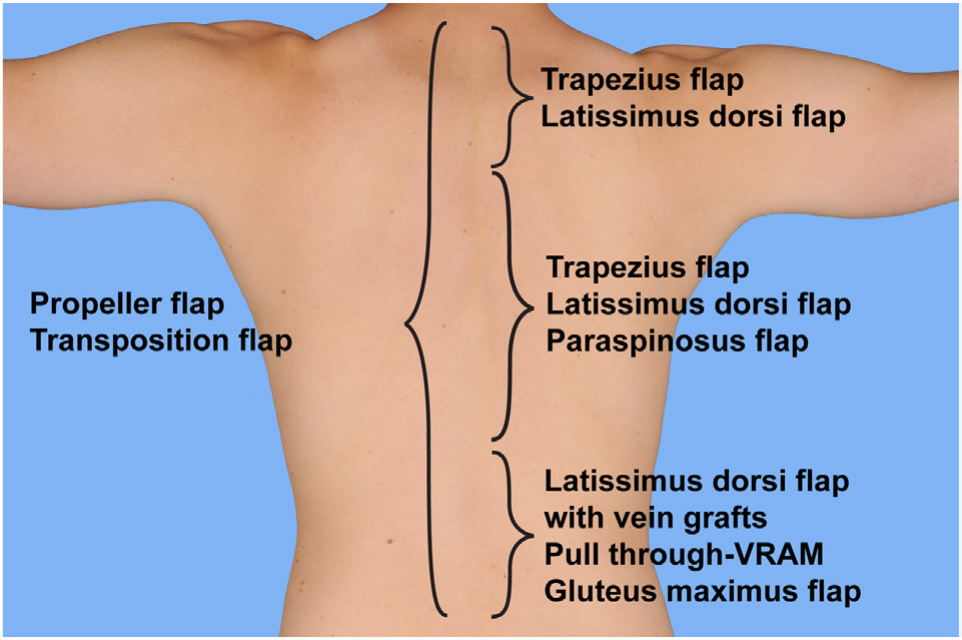
**Selection of flap based on anatomical location**. Proposed algorithm for selection of the flaps to cover posterior trunk defects.

Related to the anatomic region, several flaps have proven to be of value in reconstructing the defect. It goes without saying that the flap selection has to be adjusted to the encountered situation, surrounding tissue, and abilities and experience of the surgeon. The proposed algorithm therefore provides a rough guideline that needs to be adapted. Supposing high quality tissue in the vicinity, closure of small defects is possible with transposition or propellerflaps in all regions of the posterior trunk. In addition, the following flaps may be utilized (likewise in combination) according to the anatomic region (Figure 7; Table 1).

**Table 1:** **Options for posterior trunk defects related to the anatomic region**.

Upper defects	Propeller-/transposition flaps Trapezius flap Latissimus dorsi flap
Middle defects	Propeller-/transposition flaps Trapezius flap Latissimus dorsi flap Paraspinosus flap
Lower defects	Propeller-/transposition flaps Latissimus dorsi flap with vein grafts Pull-through VRAM Gluteus maximus flap Free flap

### Upper Defects

Given the high traction forces in the neck and shoulder region, soft-tissue reconstruction may be challenging. The majority of defects may be closed with trapezius flaps. In case the trapezius muscle is damaged or absent due to previous surgery, defects in that region may also be treated with a latissimus dorsi flap.

### Middle Defects

In the upper-thoracic region, defect coverage is possible with both, the latissimus dorsi and trapezius flap. Given the great reliability and our substantial experience, we prefer working with the latissimus dorsi flap, if possible. Both flaps can be dissected as a muscle-only flap or myocutaneous flap. If the defect requires coverage of larger areas, they might also be combined.

### Lower Defects

In the thoracolumbar and lumbar region, therapeutic options are often more complex. The extend of the latissimus dorsi flap is insufficient to reach the defect and may only be of value if combined with interposition of vein grafts, although the patient has to be in the prone position or air-fluidized bed in the early postoperative phase. According to our experience, we advise against latissimus dorsi turn-over flaps. An alternative option is provided with the pull-through VRAM in the sacral region. Additional therapeutic options are represented by paraspinous muscle flaps, gluteus maximus flaps, and microvascular free flaps, anastomosed to perforators, the superior gluteal arteries, or elongated with vein grafts.

## Summary

Given the special anatomy with few options for microvascular recipient vessel, soft-tissue defects at the posterior trunk may represent a challenge to reconstructive surgery. However, the majority of defects can be closed with pedicled flaps. If basic surgical principles such as radical debridement and removal of infected hardware are followed, good reconstructive results may be obtained with the presented methods.

## Author Contributions

All authors listed, have made substantial, direct, and intellectual contribution to the work, and approved it for publication.

## Conflict of Interest Statement

The authors declare that the research was conducted in the absence of any commercial or financial relationships that could be construed as a potential conflict of interest. The reviewers (EC and AH) and handling Editor declared their shared affiliation, and the handling Editor states that the process nevertheless met the standards of a fair and objective review.

## References

[1] Behr B Hirsch T Goertz O Ring A Lehnhardt M Daigeler A. [Therapeutic options for reconstruction of the dorsal trunk wall]. Handchir Mikrochir Plast Chir (2014) 46(2):90–6. 10.1055/s-0034-1370994 24777458

[2] Hallock GG. Reconstruction of posterior trunk defects. Semin Plast Surg (2011) 25(1):78–85. 10.1055/s-0031-1275174 22294946 PMC3140241

[3] Mathes DW Thornton JF Rohrich RJ. Management of posterior trunk defects. Plast Reconstr Surg (2006) 118(3):73e–83e. 10.1097/01.prs.0000233130.93861.15 16932161

[4] Kroll SS Rosenfield L. Perforator-based flaps for low posterior midline defects. Plast Reconstr Surg (1988) 81(4):561–6. 10.1097/00006534-198804000-00012 3279442

[5] Hamdi M Stillaert FB. Pedicled perforator flaps in the trunk. Clin Plast Surg (2010) 37(4):655–65. 10.1016/j.cps.2010.06.004 20816520

[6] Koshima I Moriguchi T Soeda S Kawata S Ohta S Ikeda A. The gluteal perforator-based flap for repair of sacral pressure sores. Plast Reconstr Surg (1993) 91(4):678–83. 10.1097/00006534-199304000-00017 8446721

[7] Roche NA Van Landuyt K Blondeel PN Matton G Monstrey SJ. The use of pedicled perforator flaps for reconstruction of lumbosacral defects. Ann Plast Surg (2000) 45(1):7–14. 10.1097/00000637-200045010-00002 10917091

[8] Pallua N Magnus Noah E. The tunneled supraclavicular island flap: an optimized technique for head and neck reconstruction. Plast Reconstr Surg (2000) 105(3):842–51. 10.1097/00006534-200003000-00003 10724241

[9] Su T Pirgousis P Fernandes R. Versatility of supraclavicular artery island flap in head and neck reconstruction of vessel-depleted and difficult necks. J Oral Maxillofac Surg (2013) 71(3):622–7. 10.1016/j.joms.2012.07.005 22921751

[10] Ramasastry SS Schlechter B Cohen M. Reconstruction of posterior trunk defects. Clin Plast Surg (1995) 22(1):167–85. 7743704

[11] Mathes SJ Nahai F Reconstructive Surgery: Principles, Anatomy and Technique. Edimburgh: Churchill Livingstone (1997).

[12] Mathes SJ Stevenson TR. Reconstruction of posterior neck and skull with vertical trapezius musculocutaneous flap. Am J Surg (1988) 156(4):248–51. 10.1016/S0002-9610(88)80284-8 3177743

[13] Disa JJ Smith AW Bilsky MH. Management of radiated reoperative wounds of the cervicothoracic spine: the role of the trapezius turnover flap. Ann Plast Surg (2001) 47(4):394–7. 10.1097/00000637-200110000-00006 11601574

[14] Chun JK Lynch MJ Poultsides GA Distal trapezius musculocutaneous flap for upper thoracic back wounds associated with spinal instrumentation and radiation. Ann Plast Surg (2003) 51(1):17–22. 10.1097/01.SAP.0000054242.00928.06 12838120

[15] Stevenson TR Rohrich RJ Pollock RA Dingman RO Bostwick J III. More experience with the “reverse” latissimus dorsi musculocutaneous flap: precise location of blood supply. Plast Reconstr Surg (1984) 74(2):237–43. 10.1097/00006534-198408000-00011 6463148

[16] Hallock GG. An extended latissimus dorsi “non-free” flap. Br J Plast Surg (1987) 40(5):516–7. 10.1016/S0007-1226(87)90085-3 3676582

[17] Wilhelmi BJ Snyder N Colquhoun T Hadjipavlou A Phillips LG. Bipedicle paraspinous muscle flaps for spinal wound closure: an anatomic and clinical study. Plast Reconstr Surg (2000) 106(6):1305–11. 10.1097/00006534-200011000-00011 11083560

[18] Daigeler A Simidjiiska-Belyaeva M Drucke D Goertz O Hirsch T Soimaru C The versatility of the pedicled vertical rectus abdominis myocutaneous flap in oncologic patients. Langenbecks Arch Surg (2011) 396(8):1271–9. 10.1007/s00423-011-0823-6 21779830

[19] Koh PK Tan BK Hong SW Tan MH Tay AG Song C The gluteus maximus muscle flap for reconstruction of sacral chordoma defects. Ann Plast Surg (2004) 53(1):44–9. 10.1097/01.sap.0000106423.87203.a3 15211198

[20] Park S Koh KS. Superior gluteal vessel as recipient for free flap reconstruction of lumbosacral defect. Plast Reconstr Surg (1998) 101(7):1842–9. 10.1097/00006534-199806000-00010 9623825

[21] Few JW Marcus JR Lee MJ Ondra S Dumanian GA. Treatment of hostile midline back wounds: an extreme approach. Plast Reconstr Surg (2000) 105(7):2448–51. 10.1097/00006534-200006000-00022 10845300

[22] Garvey PB Rhines LD Dong W Chang DW. Immediate soft-tissue reconstruction for complex defects of the spine following surgery for spinal neoplasms. Plast Reconstr Surg (2010) 125(5):1460–6. 10.1097/PRS.0b013e3181d5125e 20134362

[23] Weinstein MA McCabe JP Cammisa FP Jr. Postoperative spinal wound infection: a review of 2,391 consecutive index procedures. J Spinal Disord (2000) 13(5):422–6. 10.1097/00002517-200010000-00009 11052352

